# Epidural analgesia during esophagectomy and esophageal cancer prognosis: A population‐based nationwide study in Finland

**DOI:** 10.1111/aas.70016

**Published:** 2025-03-11

**Authors:** Pia H. Petäjäkangas, Olli Helminen, Mika Helmiö, Heikki Huhta, Raija Kallio, Vesa Koivukangas, Arto Kokkola, Simo Laine, Elina Lietzen, Sanna Meriläinen, Vesa‐Matti Pohjanen, Tuomo Rantanen, Ari Ristimäki, Jari V. Räsänen, Juha Saarnio, Eero Sihvo, Vesa Toikkanen, Tuula Tyrväinen, Antti Valtola, Joonas H. Kauppila

**Affiliations:** ^1^ Surgery Research Unit Oulu University Hospital and University of Oulu Oulu Finland; ^2^ The Division of Digestive Surgery and Urology Turku University Hospital and University of Turku Turku Finland; ^3^ Department of Oncology and Radiotherapy Oulu University Hospital Oulu Finland; ^4^ Department of Surgery University of Helsinki and Helsinki University Hospital Helsinki Finland; ^5^ Cancer and Translational Medicine Research Unit, Medical Research Center Oulu University of Oulu and Oulu University Hospital Oulu Finland; ^6^ Department of Surgery University of Eastern Finland and Kuopio University Hospital Kuopio Finland; ^7^ Department of Pathology and HUSLAB University of Helsinki and Helsinki University Hospital Helsinki Finland; ^8^ Applied Tumor Genomics Research Program, Research Programs Unit University of Helsinki Helsinki Finland; ^9^ Department of Oesophageal and General Thoracic Surgery Helsinki University Hospital and University of Helsinki Helsinki Finland; ^10^ Department of Surgery Central Finland Central Hospital Jyväskylä Finland; ^11^ Department of Cardiothoracic Surgery, Heart Center Tampere University Hospital and University of Tampere Tampere Finland; ^12^ Department of Gastroenterology and Alimentary Tract Surgery Tampere University Hospital Tampere Finland; ^13^ Upper Gastrointestinal Surgery, Department of Molecular Medicine and Surgery Karolinska Institutet Stockholm Sweden

**Keywords:** epidural analgesia, esophageal cancer, esophagectomy, mortality, prognosis

## Abstract

**Background:**

The use of epidural analgesia has been proposed to improve the prognosis of esophageal cancer by attenuating the stress response and being less immunosuppressive than opioids. This study aims to evaluate the association, if any, between non‐epidural pain management compared to epidural analgesia during minimally invasive or open esophagectomy and esophageal cancer prognosis.

**Materials and Methods:**

This was a population‐based nationwide retrospective cohort study in Finland, using the Finnish National Esophago‐Gastric Cancer Cohort (FINEGO). Esophagectomy patients with epidural and no epidural analgesia were compared. Multivariable Cox regression provided hazard ratios (HR) with 95% confidence intervals (CI) non‐epidural pain management compared to epidural analgesia, adjusted for the calendar period of surgery, sex, age, comorbidity (Charlson Comorbidity Index), tumor stage, tumor histology, neoadjuvant therapy, type of surgery, and esophageal cancer surgery volume.

**Results:**

After exclusions, there were 1381 patients available with information on epidural analgesia. Of these, 969 (70.2%) were men and 832 (60.2%) had esophageal adenocarcinoma. After adjustment for confounding factors, non‐epidural pain management was not associated with higher 90‐day mortality (HR 1.022 95% CI 0.582–1.794), overall mortality up to 5 years (HR 1.156 95% CI 0.909–1.470), nor with 5 years cancer‐specific mortality (HR 1.134 95% CI 0.884–1.456) compared to epidural analgesia.

**Conclusion:**

Although the point estimates may hint at a potentially improved prognosis associated with epidural use, this population‐based nationwide study suggests no statistically significant association between epidural analgesia during esophagectomy and esophageal cancer prognosis.

**Editorial Comment:**

This large esophagectomy (cancer) cohort in Finland was used to compare those who received epidural analgesia with those who did not for associations with late mortality in a retrospective analysis and where anesthesia and analgesia treatments were not controlled. The findings showed that when other recognized risks for mortality were taken into account, there was not a meaningful difference in relative risk for late mortality related to the presence or absence of epidural analgesia, though the analgesia treatments were not randomly allocated. These results do not rule out associations of analgesia choice with other outcomes that might be important to patients.

## 1 INTRODUCTION

Esophageal cancer is the sixth most common cause of cancer‐related death globally. The overall 5‐year survival is less than 20%.^1^ Multimodality treatment consisting of neoadjuvant therapy and surgery is the cornerstone of esophageal cancer treatment. The 5‐year survival of surgically treated esophageal cancer patients is up to 40%–50%.^2^

The use of epidural analgesia in esophageal cancer surgery has previously been associated with beneficial outcomes, for example, reducing the risk of postoperative complications such as decrease in pulmonary complications,^3^ anastomotic leaks,^4^, ^5^ 90‐day mortality,^3^, ^6^ and long‐term mortality.^6^ Epidural analgesia may attenuate the physiological stress response to surgery, causing less immunosuppression compared to opioids.^7^ This could potentially lead to lower recurrence rates^4^ and subsequent lower mortality. A previous study from the United States (*n* = 1921) suggested an association between the use of epidural analgesia and lower mortality after adjusting for confounders, including surgical approach and tumor stage.^6^ However, there is a lack of evidence on the use of epidural analgesia in relation to long‐term prognosis after esophageal cancer surgery. Based on the literature, it is hypothesized that epidural analgesia is associated with improved survival compared to no epidural analgesia. The knowledge of the short‐ and long‐term benefits of the use of epidural analgesia is still needed.

The present study aimed to assess the association, if any, between the use of epidural analgesia and prognosis after minimally invasive or open esophageal cancer surgery. It was hypothesized that non‐epidural pain management is associated with decreased survival compared to epidural analgesia.

## 2 METHODS

### 2.1 Study design

This was a population‐based nationwide retrospective cohort study based on the Finnish National Esophago‐Gastric Cancer Cohort (FINEGO), which is described in detail in a separate study protocol publication.^8^ The esophageal cancer cohort includes patients that underwent esophageal resection in Finland between the years 1987 and 2016.^9^ For this study, patients with missing information on the method of analgesia were excluded. The study was approved by all relevant government bodies and hospital districts, as well as the Ethical Committee of Northern Ostrobothnia (EETMK 115/2016).

### 2.2 Data sources

The patients were identified from the Finnish Cancer Registry and Patient Registry, which are highly complete for esophageal cancer diagnosis.^10^ The information for defining the calendar year of surgery, annual hospital volume of cancer surgery, and patient age, sex, and the recent and updated version of the Charlson Comorbidity Index (excluding esophageal cancer under treatment) was retrieved from the same registries.^11^ Furthermore, medical, pathology, and surgical records were retrieved for the identified patients from the respective hospitals and centrally evaluated for a number of clinical and medical variables by expert upper gastrointestinal surgeons and study nurses, including epidural use, tumor histology, tumor stage according to the 8th edition of TNM classification,^12^ neoadjuvant therapy, and type of surgery (minimally invasive, or open). The data collection and variable definitions were decided upon by a consensus in the collaborative group, which are described in a separate study protocol.^8^ Statistics Finland provided for mortality with 100% complete follow‐up data until December 31, 2019, and until December 31, 2018 for causes of death.

### 2.3 Exposure (epidural analgesia)

The exposure of this study was the type of analgesia (opioid analgesia vs. epidural analgesia). Non‐epidural pain management was defined as analgesia without the use of epidural analgesia, most commonly based on opioids. Epidural analgesia included the use of mainly epidural pain management during and immediately after surgery, including epidural local anesthetic with or without opioids.

### 2.4 Outcome

The primary outcome was overall all‐cause mortality calculated from the date of surgery until death or December 31, 2019. Secondary outcomes were 90‐day all‐cause mortality, 5‐year all‐cause mortality, and 5‐year cancer‐specific mortality, calculated from the date of surgery until the end of the specified follow‐up time (90 days or 5 years after surgery), death, or December 31, 2019 (December 31, 2018 for cancer‐specific mortality), whichever occurred first.

### 2.5 Statistical analysis

All analyses were done according to an a priori study protocol. The analysis plan has not been registered in an online database. Multivariable Cox regression provided hazard ratios (HR) with 95% confidence intervals (CI). The crude model was not adjusted for confounders, but model 2 was adjusted for confounding variables selected based on their known associations with esophageal cancer mortality, surgical risk, or potentially associated with epidural use. The confounders included: (1) calendar period of surgery (1987–1996, 1997–2006, or 2007–2016), (2) sex (male, or female), (3) comorbidity (Charlson Comorbidity Index score 0, 1, or ≥2), (4) tumor stage (0–I, II, III, or IV), (5) tumor histology (adenocarcinoma, or squamous cell carcinoma), (6) neoadjuvant therapy (yes, or no), (7) type of surgery (minimally invasive, or open‐surgery), and 8) hospital volume (in tertiles: ≤25, 26–72, or 73–141 per 4 years). For missing data (up to 10.4% [144/1381] patients had missing data on tumor stage, histology, and/or neoadjuvant therapy), both complete case analysis and multiple imputation were conducted. Imputation variables included all confounding variables categorized as above and all‐cause mortality. There were 20 imputed datasets, and fully conditional specification was used under the assumption that the data were missing at random. As the results using complete case analysis and multiple imputation were similar, only HRs and 95% CIs from the multiple imputation are presented. To exclude the potential selection bias due to missing records on analgesia use during the first two periods, a post hoc sensitivity analysis was done. The analysis included only patients treated during the last time period, where there were only eight missing patient records and 17 (2.5%) patients with missing epidural use information, adjusted for the confounders above. All statistical analyses were done by using IBM SPSS 26 (Armonk, NY).

## 3 RESULTS

### 3.1 Patients

A total of 2045 patients with esophagectomy and a cancer diagnosis were identified from the national registries. Of these, 1568 patients' records were available. A curative‐intent esophagectomy for cancer could be confirmed for 1456 patients. Analgesia information was available for 1381 patients who were included in this study. There were more men (*n* = 969, 70.2%) and 832 (60.2%) had adenocarcinoma.

The minority (*n* = 287, 20.8%) underwent minimally invasive surgery (no robot‐assisted surgeries, and of which 59 were hybrid procedures: 34 with thoracotomy‐laparoscopy and 25 with thoracoscopy‐laparotomy) and 1243 (90.0%) had epidural analgesia during or after surgery (Table 1). The 90‐day mortality was 14.5% in the non‐epidural pain management group and 8.2% in the epidural analgesia group. The 5‐year survival was 29.3% in the non‐epidural pain management group and 45.5% in the epidural analgesia group.

**TABLE 1:** Characteristics of the 1381 patients who underwent esophagectomy for cancer, stratified by the type of analgesia.

	Type of analgesia		
	Non‐epidural pain management	Epidural analgesia	Total
Total	138 (10.0)	1243 (90.0)	1381 (100.0)
Calendar period			
1987–1996	88 (63.8)	195 (15.7)	283 (20.5)
1997–2006	24 (17.4)	399 (32.1)	423 (30.6)
2007–2016	26 (18.8)	649 (52.2)	675 (48.9)
Sex			
Female	60 (43.5)	352 (28.3)	412 (29.8)
Male	78 (56.5)	891 (71.7)	969 (70.2)
Age at surgery			
Median (IQR)	68 (42–87)	64 (26–86)	66 (26–87)
Comorbidity			
0	94 (68.1)	785 (63.2)	879 (63.6)
1	24 (17.4)	317 (25.5)	341 (24.7)
2 or more	20 (14.5)	141 (11.3)	161 (11.7)
Tumor stage			
0‐I	27 (19.6)	361 (29.0)	388 (28.1)
II	32 (23.2)	210 (16.9)	242 (17.5)
III	57 (41.3)	462 (37.2)	519 (37.6)
IV	13 (9.4)	136 (10.9)	149 (10.8)
Missing	9 (6.5)	74 (6.0)	83 (6.0)
Tumor histology			
Adenocarcinoma	58 (42.0)	774 (62.2)	832 (60.2)
Squamous cell	75 (54.4)	421 (33.9)	461 (33.4)
Missing	5 (3.6)	48 (3.9)	88 (6.4)
Neoadjuvant therapy			
No	118 (85.5)	767 (61.7)	885 (64.1)
Yes	18 (13.0)	470 (37.8)	488 (35.3)
Missing	2 (1.5)	6 (0.5)	8 (0.6)
Type of surgery			
Open	128 (92.8)	966 (77.7)	1094 (79.2)
Minimally invasive	10 (7.2)	277 (22.3)	287 (20.8)
Annual hospital volume			
Lowest tertile	103 (74.6)	347 (27.9)	450 (32.6)
Middle tertile	23 (16.7)	442 (35.6)	465 (33.7)
Highest tertile	12 (8.7)	454 (36.5)	466 (33.7)

### 3.2 5‐year all‐cause mortality

There were 768 deaths during the 5‐year follow‐up, of which 702 (91.4%) were due to esophageal cancer. Non‐epidural pain management analgesia was associated with increased 5‐year all‐cause mortality in crude (HR 1.611 95% CI 1.302–1.994), compared to epidural analgesia. After adjustment for confounders, this association was mitigated (HR 1.156 95% CI 0.909–1.470) (Table 2). The sensitivity analysis of the more recent period of 2007–2016 showed no association between analgesia and mortality (adjusted HR 0.675 95% CI 0.362–1.260) (Table 2).

**TABLE 2:** Risk of all‐cause mortality during 5‐year follow‐up without epidural analgesia compared with epidural analgesia in patients undergoing esophagectomy for esophageal cancer, presented as hazard ratios (HR) and 95% confidence intervals (CIs).

Model	N	No epidural analgesia	Epidural analgesia
		HR (95% CI)	HR (95% CI)
All patients			
Crude	1381	1.611 (1.302–1.994)	1.00 (reference)
Adjusted^a^	1381	1.156 (0.909–1.470)	1.00 (reference)
Subgroup analysis including years 2007–2016			
Crude	675	0.811 (0.445–1.480)	1.00 (reference)
Adjusted^a^	675	0.675 (0.362–1.260)	1.00 (reference)

^a^ Adjusted for confounding variables: time period, age, sex, comorbidity, tumor histology, tumor stage, neoadjuvant therapy, type of surgery, and annual hospital volume.

### 3.3 Secondary outcomes

Similarly to the main outcome, non‐epidural pain management was associated with increased overall all‐cause mortality rates in crude (HR1. 427 95% CI 1.178–1.729), but not in adjusted analysis (HR 1.105 95% CI 0.892–1.368), compared to the use of epidural analgesia (Table 2). No association between epidural use and 90‐day mortality, nor 5‐year cancer‐specific mortality was present in the adjusted analyses (Table 3). In the subgroup analysis of patients operated during years 2007–2016, there were no statistically significant differences between the analgesia and for any mortality outcomes (Table 3).

**TABLE 3:** Risk of 90‐day all‐cause mortality, overall all‐cause mortality, and 5‐year cancer‐specific mortality without epidural analgesia compared with epidural analgesia in patients undergoing esophagectomy for esophageal cancer, presented as hazard ratios (HR) and 95% confidence intervals (CI).

Model	*N*	No epidural analgesia	Epidural analgesia
		HR (95% CI)	HR (95% CI)
90‐day all‐cause mortality			
Crude	1381	2.053 (1.266–3.330)	1.00 (reference)
Adjusted^a^	1381	1.022 (0.582–1.794)	1.00 (reference)
Subgroup analysis including years 2007–2016			
Crude	675	N/A^b^	1.00 (reference)
Adjusted^a^	675	N/A^b^	1.00 (reference)
Overall all‐cause mortality			
Crude	1381	1.427 (1.178–1.729)	1.00 (reference)
Adjusted^a^	1381	1.105 (0.892–1.368)	1.00 (reference)
Subgroup analysis including years 2007–2016			
Crude	675	0.826 (0.475–1.437)	1.00 (reference)
Adjusted^a^	675	0.692 (0.389–1.231)	1.00 (reference)
Cancer‐specific mortality up to 5 years			
Crude	1381	1.642 (1.317–2.046)	1.00 (reference)
Adjusted^a^	1381	1.134 (0.884–1.456)	1.00 (reference)
Subgroup analysis including years 2007–2016			
Crude	675	0.668 (0.331–1.349)	1.00 (reference)
Adjusted^a^	675	0.564 (0.274–1.161)	1.00 (reference)

^a^ Adjusted for confounding variables: time period, age, sex, comorbidity, tumor histology, tumor stage, neoadjuvant therapy, type of surgery, and annual hospital volume.

^b^ Not calculated due to no events in the no epidural analgesia group.

### 3.4 Survival over time

A post hoc evaluation of survival over time shows that during 1987–1996, the 5‐year survival was 29.7%, improving to 43.0% in 1997–2006 and to 50.5% during 2007–2016. The use of epidural analgesia increased over time (Table 1), as did the 5‐year survival in those receiving epidural analgesia, being 34.4% in 1987–1996, 43.4% during 1997–2006, and 50.3% during 2007–2016 compared to 19.3% in 1987–1996, 37.5% in 1997–2006, and 55.9% in those not receiving epidural analgesia.

## 4 DISCUSSION

The results of the present study suggest that the use of epidural analgesia is associated with a better prognosis after esophageal cancer surgery. After adjustment for confounders, this effect was mitigated and therefore, this effect seems to be largely explained by confounding factors, but the survival benefit from epidural analgesia cannot be ruled out.

The strengths of this study include the population‐based nationwide design reducing selection bias and the large size of the cohort, allowing robust estimates in the main analysis. The proportion of patients with completely missing records and missing information on the use of epidural analgesia, mostly during the earlier years of the study, might have caused some selection bias. This potential bias was taken into account by adjusting for the year of surgery and conducting a sensitivity analysis including only the latest time period where the records and the data were highly complete, in which the results were not different from the main analysis. As epidural analgesia is routinely used, the reasons for not using epidural analgesia could cause bias. Paravertebral block analgesia was not used in Finland during the study period. The insertion could have been unsuccessful due to, for example, obesity, previous spine surgery or spine deformities, or due to contraindications such as anticoagulation or other contraindications such as a history of intracranial hemorrhage. There may also be other clinical reasons why epidural analgesia was not used, for example, related to the physical condition of the patient or patient preferences, causing potential confounding by indication. Therefore, those in the non‐epidural pain management group could have an increased risk of long‐term mortality not adjusted by the Charlson Comorbidity Index. This bias should, however, skew the result toward improved outcomes after epidural analgesia instead of no difference, as observed in the current study. The main weakness of the study is the lack of information on some important variables, including the duration of the epidural analgesia, drugs used in epidural infusions, and data on opioid use, which may bias the results toward null as presumably all patients received opioids during or after their hospitalization. Due to the low number of patients in the non‐epidural pain management group, low statistical power led to wide confidence intervals and might result in chance findings, especially in subgroup analysis of time periods. Missing confounder data were handled with multiple imputation. Additionally, the complete ascertainment of known preoperative prognostic variables allowed adjustment for all known confounding variables. The follow‐up was complete with no missing outcome data.

There are only a few previous studies examining the use of epidural analgesia in relation to long‐term prognosis in esophageal cancer. A prospective study from the United Kingdom (*n* = 140) suggested that the use of epidural analgesia is associated with a lower risk of esophageal cancer recurrence within 2 years (HR 0.34 95% CI [0.16–0.75]) and better overall survival (epidural vs. without: 75%/67%) after 2 years.^13^ However, that study had a small sample size and originated from a single center. A retrospective cohort study from the United States (*n* = 1169) suggested that the use of epidural analgesia is associated with lower 90‐day mortality (5.6% vs. 8.9%) and better 5‐year survival (33.5% vs. 26.5%), compared to no epidural analgesia.^6^ However, that study did not include patients with minimally invasive surgery or adjust for neoadjuvant therapy. For short‐term outcomes, a German cohort study (*n* = 335) of patients undergoing esophagectomy for esophageal cancer suggested that epidural analgesia reduced the incidence of postoperative pulmonary complications and was associated with lower 90‐day mortality, compared to no epidural analgesia.^3^ A Japanese study of 12,688 patients undergoing minimally invasive esophagectomy suggested that epidural analgesia was associated with lower in‐hospital mortality, compared to no epidural analgesia, but did not evaluate long‐term outcomes.^14^ The current study was adjusted for all important potential confounders, some of which were not taken into account in the aforementioned studies. In the present study, the use of epidural analgesia was not associated with short‐ or long‐term prognosis after adjustment for confounding. The point estimates in the post hoc sensitivity analysis, including only patients from the most recent period, suggested potentially lower mortality in patients without epidural analgesia, contradicting the study hypothesis. However, none of the differences were statistically significant after adjustment, and the power in the analysis was low. Taken together, the present analysis indicates that previous studies suggesting better survival with epidural analgesia may be confounded. The survival benefit of epidural analgesia seems to be modest at best, or there may not be any such benefit.

While epidural analgesia may not be relevant for survival outcomes after esophagectomy, the use of epidural analgesia has other major benefits, including stable pain management, potentially reduced incidence of postoperative pulmonary complications,^4^, ^15^ and anastomotic leakage.^14^ The use of epidural pain management may also alleviate the physiological stress response to surgery,^7^, ^15^ cause less immunosuppression,^16^ maybe even less than opioids,^17^ and reduce the risk of developing chronic post‐thoracotomy pain with a minimal risk of motor block.^15^ Therefore, with current evidence, epidural anesthesia should remain as an integral part of postoperative care after esophageal cancer surgery, although no association was seen with short‐ or long‐term mortality outcomes. In the future, well‐powered or randomized studies are needed to test the benefit of epidural analgesia regarding complications, as well as to validate the findings of the current study. Whether different anesthetics and additives in the epidural analgesia solution have differential effects on short‐ or long‐term outcomes after esophagectomy remain to be explored.

In conclusion, this population‐based nationwide study suggests that the use of epidural analgesia is not statistically significantly associated with decreased mortality after esophageal cancer surgery. However, improved survival related to epidural analgesia cannot be ruled out, and additional studies to confirm these findings are needed.

## AUTHOR CONTRIBUTIONS

**OH, MH, HH, RK, VK, AK, SL, EL, SM, V‐MP, TR, AR, JVR, JS, ES, VT, TT, AV, JHK**: Study conception, data collection and curation, critical review of the manuscript and approval of the submitted manuscript. **PP**: Study conception, data collection and curation, critical review of the manuscript, approval of the submitted manuscript, and writing up of the first draft of the paper.

## Data Availability

Due to current legislation, the original data cannot be made publicly available. The data may be accessed after this has been approved by the relevant government and institutional bodies, and the authors can help in this process. The study or statistical analysis were not pre‐registered in any independent institutional registry.
